# Risk of hyperglycemia with different statins by type and dose: a network meta-analysis and real-world pharmacovigilance study

**DOI:** 10.3389/fphar.2026.1867316

**Published:** 2026-10-02

**Authors:** Qingxia Xue, Feixian Wang, Yanyun Zhang, Lu Chen

**Affiliations:** 1 Department of Pharmacy, Yantai Yuhuangding Hospital, Yantai, Shandong, China; 2 Department of Physical Examination Center, Yantai Yuhuangding Hospital, Yantai, Shandong, China

**Keywords:** disproportionality analysis, hyperglycemia-related adverse events, meta-analysis, pharmacovigilance, statin

## Abstract

**Background:**

The potential diabetogenic effects of statins remain controversial, particularly with respect to differences across statin types and doses.

**Methods:**

We conducted a network meta-analysis (NMA) of randomized controlled trials (RCTs) to assess the comparative effects of different statins and doses on hyperglycemia-related outcomes. The NMA was performed within a Bayesian framework. PubMed, Embase, the Cochrane Library, and ClinicalTrials.gov were systematically searched from inception to April 2025. Data extraction and verification were independently conducted by two investigators. Outcomes included risk of new-onset diabetes mellitus (NODM), fasting plasma glucose (FPG), 2-h postprandial plasma glucose, and glycated hemoglobin (HbA1c). Separately, we conducted a real-world pharmacovigilance analysis using disproportionality methods based on the FDA Adverse Event Reporting System (FAERS) to identify potential hyperglycemia-related safety signals in patients without diabetes.

**Results:**

A total of 22 RCTs, which enrolled 99,311 patients, were included. The NMA found no statistically significant differences in NODM risk among statins or compared with placebo. However, pravastatin 40 mg/day showed a numerically lower risk estimate (OR: 0.86; 95% CrI: 0.50–1.50), whereas rosuvastatin 20 mg/day and atorvastatin 80 mg/day showed relatively higher estimates. Analyses of intermediate glycemic markers, including FPG and HbA1c, revealed no significant differences across all statin regimens and placebo. In parallel, FAERS pharmacovigilance analyses identified disproportionate reporting signals for hyperglycemic events with atorvastatin (ROR: 6.11; 95% CI: 5.94–6.29), rosuvastatin (ROR: 2.44; 95% CI: 2.31–2.58), and simvastatin (ROR: 1.22; 95% CI: 1.13–1.32).

**Conclusion:**

No statistically significant differences in glycemic outcomes were observed among individual statins or compared with placebo. However, numerical variations in point estimates across randomized evidence and pharmacovigilance signals suggest possible differences in glycemic profiles among statins. Pravastatin showed numerically lower risk estimates; however, these findings were accompanied by substantial uncertainty. Further prospective studies are needed to clarify these observations.

## 1 Introduction

Statins are the first-line pharmacologic treatment for managing hypercholesterolemia in patients at risk of atherosclerotic cardiovascular (CV) disease (Baigent et al., 2005). While statin therapy confers clear survival benefits, emerging evidence suggests an unexpected association with an increased incidence of new-onset diabetes mellitus (NODM) or abnormality in blood glucose (Newman et al., 2019). Previous systematic reviews have demonstrated this risk through the quantitative synthesis of randomized evidence. For instance, a meta-analysis of 91,140 participants across 13 trials revealed that statin therapy increased diabetes incidence (odds ratio [OR]: 1.09; 95% credible intervals [CrI]: 1.02–1.17) (Sattar et al., 2010). Similarly, comparative analyses of intensive- versus moderate-dose statin therapy (median follow-up 4.9 years) showed higher diabetes rates in the intensive group (8.8% [1,449/16,408] vs. 8.0% [1,300/16,344]; OR: 1.12; 95% CrI: 1.04–1.22), corresponding to an extra two cases of diabetes mellitus per 1,000 patient-years of treatment (Preiss et al., 2011). Notably, some studies have reported no significant difference in NODM or impaired glucose metabolism for rosuvastatin (Yusuf et al., 2016) or pitavastatin (Vallejo-Vaz et al., 2015) compared with placebo or other statins. Taken together, these findings indicate substantial heterogeneity in glycemic effects across different statins and dosing regimens. Consequently, current guidelines provide limited guidance on prioritizing specific statins or doses, particularly for glucose-sensitive populations, leaving clinicians and patients uncertain about the magnitude of statins’ adverse effects on glucose metabolism.

Given the widespread and growing real-world use of statins, it is imperative to evaluate and compare the risk of diabetes associated with different statin therapies. Accordingly, this study aimed to assess the risk of statin-associated hyperglycemia by performing a network meta-analysis (NMA) of randomized controlled trials (RCTs). Additionally, we conducted a complementary pharmacovigilance analysis using the Food and Drug Administration (FDA) Adverse Event Reporting System (FAERS) to detect hyperglycemia-related signals in clinical practice. This combined approach effectively identified adverse events (AEs), as both methods complemented each other and strengthened the robustness of the evidence (Khouri et al., 2018; Dolladille et al., 2020). These findings may contribute to the evidence base for individualized statin prescribing by improving understanding of the potential glycemic effects of different statin therapies.

## 2 Methods

### 2.1 Study design

We first performed an NMA of RCTs to investigate the risk of hyperglycemia associated with different types and doses of statins. Subsequently, a retrospective data mining analysis of the FAERS database was conducted to provide complementary real-world evidence on the risk of hyperglycemia with associated statins.

### 2.2 Search strategy and selection criteria

This study was prospectively registered in PROSPERO (CRD42025640783). The NMA was performed in accordance with the PRISMA checklist (Supplementary Table S1). PubMed, Embase, the Cochrane Central Register of Controlled Trials, and ClinicalTrials.gov were searched to identify RCTs of statins (from inception to April 2025), with no language restriction. A manual search of reference lists of relevant studies was also performed to identify further eligible studies (Supplementary Table S2). RCTs were eligible for inclusion when they compared placebo- or statin-controlled (atorvastatin, rosuvastatin, pitavastatin, pravastatin, simvastatin, fluvastatin, and lovastatin), parallel arm, including participants without diabetes, and reported at least one of the main outcomes of interest: risk of NODM, fasting plasma glucose (FPG), 2-h postprandial plasma glucose, or glycated hemoglobin (HbA1c). The primary outcome of the NODM was defined as the number of non-diabetic subjects at baseline who reported with diagnoses of diabetes during the trial by a physician or commenced glucose-lowering medication during the trial. Studies with a design not reported clearly, and those involving pregnant women, subjects with acute conditions, or decompensated states of chronic diseases were excluded. Two researchers (X.Q.X. and W.F.X.) independently reviewed potentially relevant articles and abstracted necessary data, and discrepancies were resolved through consultation with a third researcher (C.L. or Z.Y.Y.).

### 2.3 Data synthesis and statistical analysis

For dichotomous outcomes, we estimated the results using odds ratio (OR) with 95% credible interval (CrI). For continuous outcomes, mean differences (MD) with 95% CrI were used. For each outcome with ≥2 studies in the network, we fitted Bayesian random-effects models with non-informative priors for treatment effects and heterogeneity, running four chains of 200,000 iterations (burn-in 100,000, thinning 10). Convergence was assessed by trace plots, the Brooks–Gelman–Rubin diagnostic (PSRF approaching 1.00), and model fit by deviance information criterion (DIC); random-effects models were preferred when appropriate. Within the Bayesian framework, all interventions were ranked using a surface-under-the-cumulative-ranking (SUCRA) curve, with values approaching 100% indicating a higher probability of being the best treatment for the outcome of interest. The Q test and I^2^ statistic were utilized to assess heterogeneity among studies, with I^2^ values over 50% and p < 0.10 suggesting substantial between-study heterogeneity. Both direct and indirect comparisons between any pair of comparators (existing closed loops) employed the node-splitting approach to examine consistency. For outcomes without adequate network structure, we performed pairwise meta-analyses only, or descriptive analyses were conducted if there was insufficient similarity information to pool the data. In the event of a disconnected network, separate analyses were performed for each sub-network, with results presented accordingly. No indirect comparisons could be made between interventions in different sub-networks. The risk of bias of the RCTs was assessed using the Cochrane Collaboration’s Risk of Bias 2 (RoB 2.0) tool. Subgroup analyses or meta-regression were assessed by baseline characteristics when sufficient information was available. Additionally, sensitivity analysis was performed by excluding studies with a high risk of bias. A comparison-adjusted funnel plot and Egger test were used to evaluate small-study effects for individual outcomes when at least ten eligible studies were available. Statistics were performed using Stata 14.0 and R 4.4.2 software. Statistical significance was set at p < 0.05.

### 2.4 Pharmacovigilance study procedure

Real-world pharmacovigilance data for hyperglycemic AEs were extracted from the FAERS database at 2004Q1–2024Q4. Reports were included if any of the following statins were listed as the primary suspect (PS) drug: atorvastatin, rosuvastatin, pitavastatin, pravastatin, simvastatin, fluvastatin, or lovastatin. Adverse events in FAERS are coded by the preferred-terms level of the *Medical Dictionary for Regulatory Activities* (MedDRA version 27.0) classification. The predefined primary endpoint was any hyperglycemic AEs, including all terms in the Standardized MedDRA Query (SMQ)—“hyperglycemic/new-onset diabetes mellitus”. Hyperglycemic AEs were grouped into predefined subcategories: mild hyperglycemia and diabetes (Supplementary Table S3).

When available, data of clinical characteristics were also collected. Before performing statistical analysis, duplicate reports were removed by reviewing the unique ID and case characteristics. To avoid bias, data mining was independently performed using the OpenVigil FDA tool. Signal detection for AEs risk was performed using disproportionality analysis based on the reporting odds ratio (ROR) and 95% confidence intervals (CI), with reports involving all other drugs in the FAERS database serving as the comparator. For ROR, the lower limit of the 95% CI of the ROR (ROR_025_) >1 with at least three cases indicated a significant risk signal. All analyses were performed using Microsoft Excel (2021), with calculations verified independently by two researchers (X.Q.X. and C.L.).

## 3 Results

### 3.1 Meta-analysis

#### 3.1.1 Literature search, study selection, and characteristics

The initial search strategy identified 9,824 records after removing duplicates, of which 22 RCTs met the inclusion criteria in the final analysis (Figure 1). The baseline characteristics of the included studies are summarized in Table 1. Rosuvastatin was used in 12 trials, atorvastatin in 7, pitavastatin in 3, pravastatin in 3, and simvastatin in 3 trials. For the NODM outcome, the included statins formed a connected treatment network. The included statin regimens were atorvastatin (10, 20, 40, and 80 mg), rosuvastatin (10, 20, and 40 mg), pravastatin (40 mg), simvastatin (40 mg), and pitavastatin (2 and 4 mg). In contrast, the analyses of HbA1c and FPG involved two disconnected networks of interventions. The first included atorvastatin (10 and 20 mg), rosuvastatin (5, 10, and 20 mg), and simvastatin (40 mg), while the second network included pitavastatin (2 and 4 mg) and pravastatin (40 mg) (Figure 2).

**FIGURE 1:**
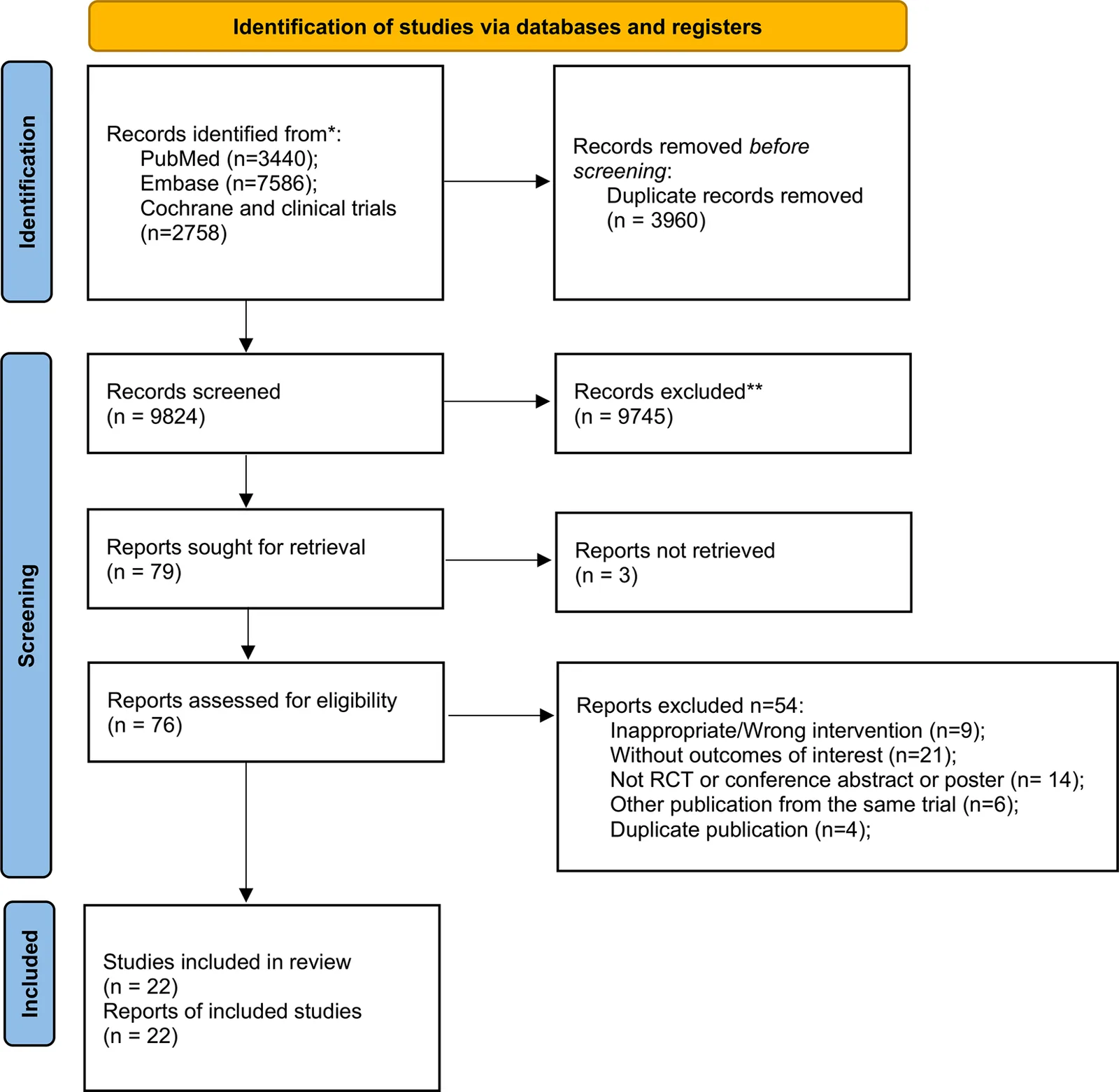
Study flow diagram of study selection process.

**TABLE 1:** Basic characteristics of the included studies.

First author (year)	Clinical study ID	Study design	T/C	Sample size (T/C)	Age (mean ± SD)	Gender (T/C, female%)	Duration of study	FPG (mg/dL) (mean ± SD)	HbA1c (%) (mean ± SD)	BMI (kg/m^2^) (mean ± SD)	Follow-up time
Freeman et al. (2001)	WOSCOPS trial	Randomized, double-blind	Pravastatin 40 mg/day Placebo	2,999/2,975	55.2 ± 5.5	0%/0%	6.1 y	84.96 ± 0.51	NR	25.9 ± 3.1	6.1 y
Collins et al. (2003)	HPS trial	Randomized	Simvastatin 40 mg/day Placebo	7,291/7,282	64.7 ± 8.1^c^	22%^c^	NR	NR	NR	NR	4.6 y
Keech et al. (2003)	LIPID trial	Randomized, double-blind	Pravastatin 40 mg/day Placebo	3,496/3,501	62 (55–67)^bc^	17%^c^	6.1 y	93.6 (88.2–99)^bc^	NR	NR	6.0 y
Keech et al. (2003)	LIPID trial	Randomized, double-blind	Pravastatin 40 mg/day Placebo	474/466	63 (55–67.5)^bc^	15%^c^	6.1 y	113.4 (111.6–118.8)^bc^	NR	NR	6.0 y
Sever et al. (2004)	ASCOT-LLA trial	Randomized, double-blind	Atorvastatin 10 mg/day Placebo	5,168 (3,910)/5,137 (3,863)	63.1 ± 8.5 63.2 ± 8.6	18.9%/18.7%	3.3y^b^	111.6 ± 37.8 111.6 ± 37.8	NR	28.6 ± 4.7 28.7 ± 4.6	3.3y^b^
Kjekshus et al. (2007)	NCT00206310	Randomized, double-blind	Rosuvastatin 10 mg/day Placebo	2,514 (1,771)/2,497 (1,763)	73 ± 7.1 73 ± 7.0	24%/24%	3.5 y	NR	NR	27 ± 4.5 27 ± 4.6	3.5 y
Chan et al. (2010)	ISRCTN32424163	Randomized, double-blind	Rosuvastatin 40 mg/day Placebo	134/135	58 ± 12.9 57.9 ± 14.3	39.5%/37%	5 y	NR	NR	27.7 ± 5.05 28.5 ± 6.21	4 y
Ridker et al. (2012)	NCT00239681	Randomized, double-blind	Rosuvastatin 20 mg/day Placebo	8,901/8,901	66.1 ± 7.64 66.1 ± 7.80	38.5%/37.9%	5 y	94 (87–102)^b^ 94 (88–102)	5.7 (5.4–5.9)^b^ 5.7 (5.5–5.9)	28.3 (25.3–32)^b^ 28.4 (25.3–32)	3.5 y
Ong et al. (2014)	TNT trial	Randomized	Atorvastatin 10 mg/day Atorvastatin 80 mg/day	3,797/3,798	60.6 ± 8.9^c^	17.4%^c^	NR	97.4 ± 0.59^c^	NR	28.10 ± 4.24^c^	4.9 y^b^
Erlandson et al. (2015)	NCT01218802	Randomized, double-blind	Rosuvastatin 10 mg/day Placebo	72/75	45.6 (41.1–51.4)^b^ 46.9 (39.2–53.6)^b^	19%/24%	3.25 y	84 (75, 95)^b^ 87 (79, 94)^b^	NR	26.6 (23.4–30.0)^b^ 27.2 (23.5–30.5)^b^	96 w
Yusuf et al. (2016)	NCT00468923	Randomized, double-blind	Rosuvastatin 10 mg/day Placebo	6,361 (5,987)/6,344 (5,987)	65.8 ± 6.4 65.7 ± 6.3	46.4%/46.1%	5.6 y	95.4 (87–106.2)^b^ 95.4 (86.4–106)^b^	NR	27.15 ± 4.78 27.07 ± 4.77	5.6 y^b^
Lee et al. (2020)	NCT02056847	Randomized, open-labeled	Pitavastatin 4 mg/day Pitavastatin 2 mg/day	153/146	57.4 ± 7.3 58.6 ± 8.0	39.87%/41.10%	12 m	107.62 ± 6.82 106.42 ± 8.74	5.88 ± 0.31 5.87 ± 0.35	25.93 ± 3.02 25.54 ± 2.90	12 m
Zheng et al. (2022)	NCT02546323	Randomized, double-blind	Rosuvastatin 20 mg/day Placebo	272/271	59.0 ± 5.2 59.7 ± 5.0	53.7%/58.3%	3.3 y	FPG≥110 mg/dL: 14.7%/11.1%	NR	25.0 ± 2.6 24.7 ± 2.7	611.8 d 613.8 d
Grinspoon et al. (2023)	NCT02344290	Randomized, quadruple	Pitavastatin 4 mg/day Placebo	3,888/3,881	50 (45–55)^b^ 50 (45–55)^b^	31.1%/31.1%	8.4 y	NR	NR	NR	5.1 y (4.3–5.9)^b^
Hong et al. (2024)	NCT02579499	Randomized, open-labeled, single-center	Rosuvastatin (10–20 mg/day) Atorvastatin (20–40 mg/day)	1,259/1,118	65 ± 10 65 ± 10	29%/28%	3 y	<99 mg/dL: 41%/37% 100–125: 51%/53% ≥126: 9%/11%	NR	24.5 ± 2.9 24.5 ± 2.7	3 y
Armitage et al. (2010)	NCT00124072	Randomized, double-blind	Simvastatin 80 mg/day Simvastatin 20 mg/day	6,031 (5,398)/6,033 (5,399)	64.2 ± 8.9 64.2 ± 8.9	19.5%/22.2%	NR	NR	NR	NR	6.7 y
Park et al. (2010)	NCT00335699	Randomized, open-labeled	Rosuvastatin 10 mg/day Atorvastatin 10 mg/day	172/178	60.49 ± 0.74^a^ 58.96 ± 0.75^a^	57.56%/62.36%	6 w	91.08 ± 0.75^a^ 93.32 ± 0.84	5.88 ± 0.04^a^ 5.86 ± 0.03	NR	6 w
Moutzouri et al. (2011)	NR	Randomized, open-labeled	Rosuvastatin 10 mg/day Simvastatin 40 mg/day	45/55	52 ± 16 58 ± 8	63%/67%	12 w	96 ± 9 92 ± 11	5.9 ± 0.1 6.0 ± 0.3	29 ± 9 29 ± 8	12 w
Koh et al. (2016)	NR	Randomized, single-blind	Rosuvastatin 5 mg/day Rosuvastatin 10 mg/day Rosuvastatin 20 mg/day Placebo	47/48/47/48	56 ± 7 58 ± 8 57 ± 7 57 ± 8	48.94%/47.92%/51.06%/52.08%	NR	98 ± 11 98 ± 11 99 ± 27 94 ± 11	5.88 ± 0.53 5.88 ± 0.54 5.84 ± 0.56 5.94 ± 0.51	25.51 ± 2.07 24.98 ± 1.97 25.64 ± 2.01 25.24 ± 2.18	2 m
Her et al. (2010)	NR	Randomized, open-label, single-center	Atorvastatin 20 mg/day Rosuvastatin 10 mg/day	25/25	63 ± 9 56 ± 10	72%/72%	8 w	101 ± 22 97 ± 10	5.89 ± 0.66 5.68 ± 0.36	25.1 ± 2.5 25.2 ± 3.5	8 w
Anagnostis et al. (2014)	NR	Randomized, open-label	Rosuvastatin 10 mg/day Atorvastatin 20 mg/day	24/28	57.4 ± 1.9 56.1 ± 2.2	83.3%/78.6%	12 w	91.0 ± 2.4 94.3 ± 2.1	5.5 ± 0.1 5.6 ± 0.1	29.7 ± 0.7 30.9 ± 0.8	12 w
Agrawal et al. (2018)	NR	Randomized, open-label	Atorvastatin 40 mg/day Atorvastatin 80 mg/day	120/120	57.48 ± 11.17 59.05 ± 10.25	26.67%/20.00%	6 m	NR	5.19 ± 0.24 5.17 ± 0.24	NR	6 m
Aberg et al. (2017)	NCT01301066	Randomized, double-blind	Pitavastatin 4 mg/day Pravastatin 40 mg/day	126/126	50.1 ± 7.5 49.2 ± 8.7	16%/12%	2.25 y	93.6 ± 0.6 95.4 ± 0.6	5.4 ± 0.4 5.5 ± 0.4	27.2 ± 4.5 28.2 ± 4.9	52 w

a: mean ± SE; b: median (Q1–Q3) or median; c: data reported for the overall study population (intervention and control groups combined).

Abbreviations: FPG, fasting plasma glucose; HbA1c, glycated hemoglobin; BMI, body mass index; T/C, treatment/control; NR, not report; d, days; w, weeks; m, months; y, years.

**FIGURE 2:**
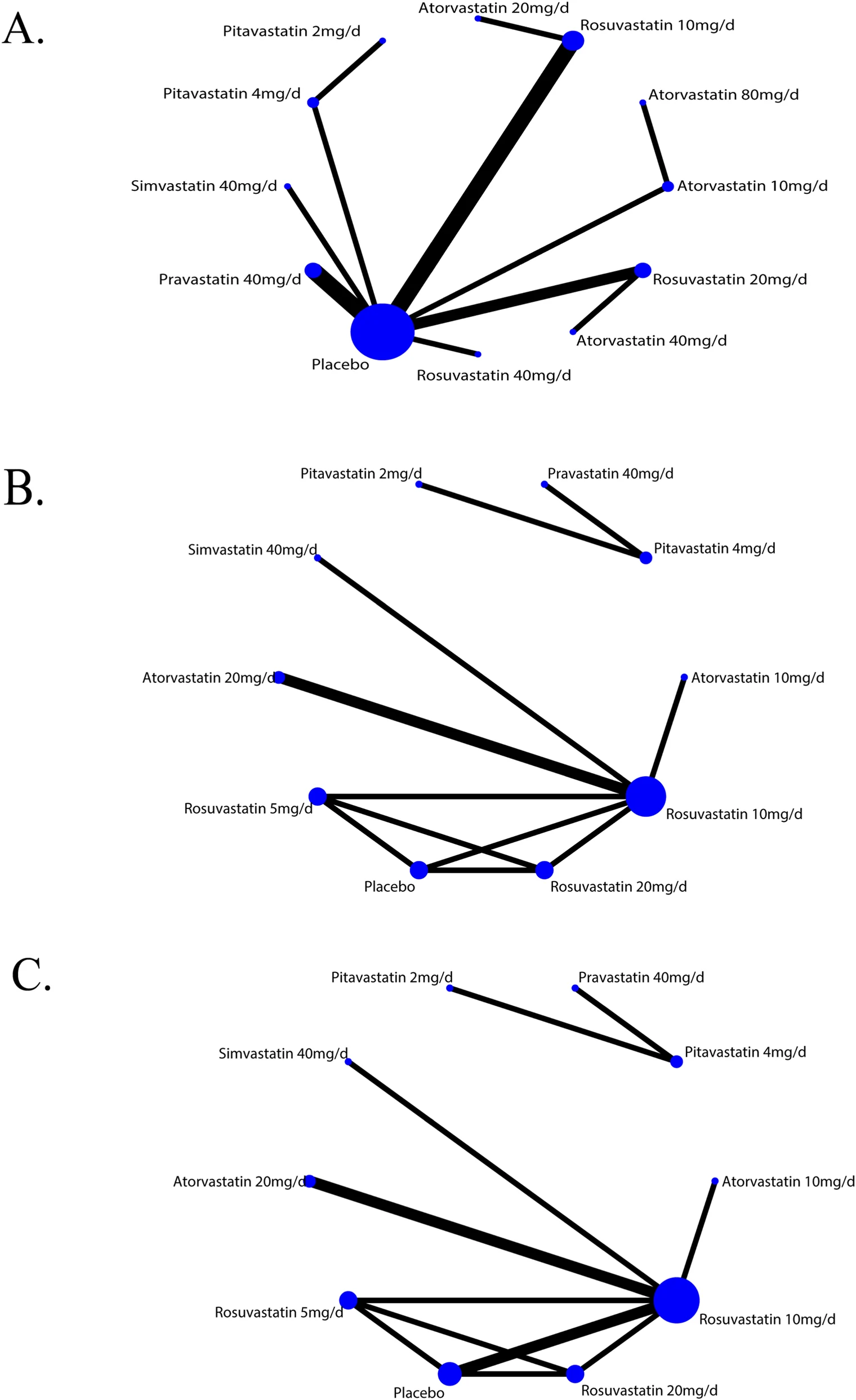
Network diagrams of comparisons on outcomes. **(A)** Risk of NODM. **(B)** Mean difference in HbA1c. **(C)** Mean difference in FPG.

#### 3.1.2 Risk of bias

RoB-2 assessment showed that 14 studies (63.6%) were evaluated as having a low risk of bias, 7 (31.8%) had some concerns, and 1 study had a high risk due to lack of allocation concealment and missing outcome data (Supplementary Figure S1).

#### 3.1.3 Primary outcome: NODM

Pooled data from 15 trials involving 98,145 patients were analyzed to evaluate the incidence of NODM (Yusuf et al., 2016; Freeman et al., 2001; Collins et al., 2003; Keech et al., 2003; Sever et al., 2004; Kjekshus et al., 2007; Chan et al., 2010; Ridker et al., 2012; Ong et al., 2014; Erlandson et al., 2015; Lee et al., 2020; Zheng et al., 2022; Grinspoon et al., 2023; Hong et al., 2024; Armitage et al., 2010). Head-to-head comparisons showed that there were no statistically significant association of different doses of statins with the risk of NODM compared with placebo or other doses of statins, excepting pitavastatin 4 mg/day, which was significantly associated with higher risk of NODM than placebo (OR: 1.35; 95% CI: 1.09–1.67) (Figure 3). However, the synthesized results of NMA demonstrated no significant differences in the risk of NODM between the different doses of statins and placebo, and no significant difference between statin doses (Figure 3). Although not statistically significant, the point estimate for pravastatin 40 mg/day was numerically lower than placebo (OR: 0.86; 95% CrI: 0.5–1.5). According to the surface under the cumulative ranking curve (SUCRA) values, pravastatin 40 mg/day ranked first, followed by rosuvastatin 10 mg/day (Supplementary Figure S3A). Due to the absence of closed loops in the network, a formal consistency assessment between direct and indirect evidence could not be performed. No publication bias was observed among the studies included for the meta-analysis of NODM in the present study (Egger’s test, P = 0.99; Supplementary Figure S4). There was no material change observed in the results by excluding the trial evaluated as having a high risk of bias. By meta-regression results, similarities in clinical characteristics (age, female ratio, and follow-up time) were observed across all included studies, suggesting that the transitivity assumption was met (Supplementary Table S4).

**FIGURE 3:**
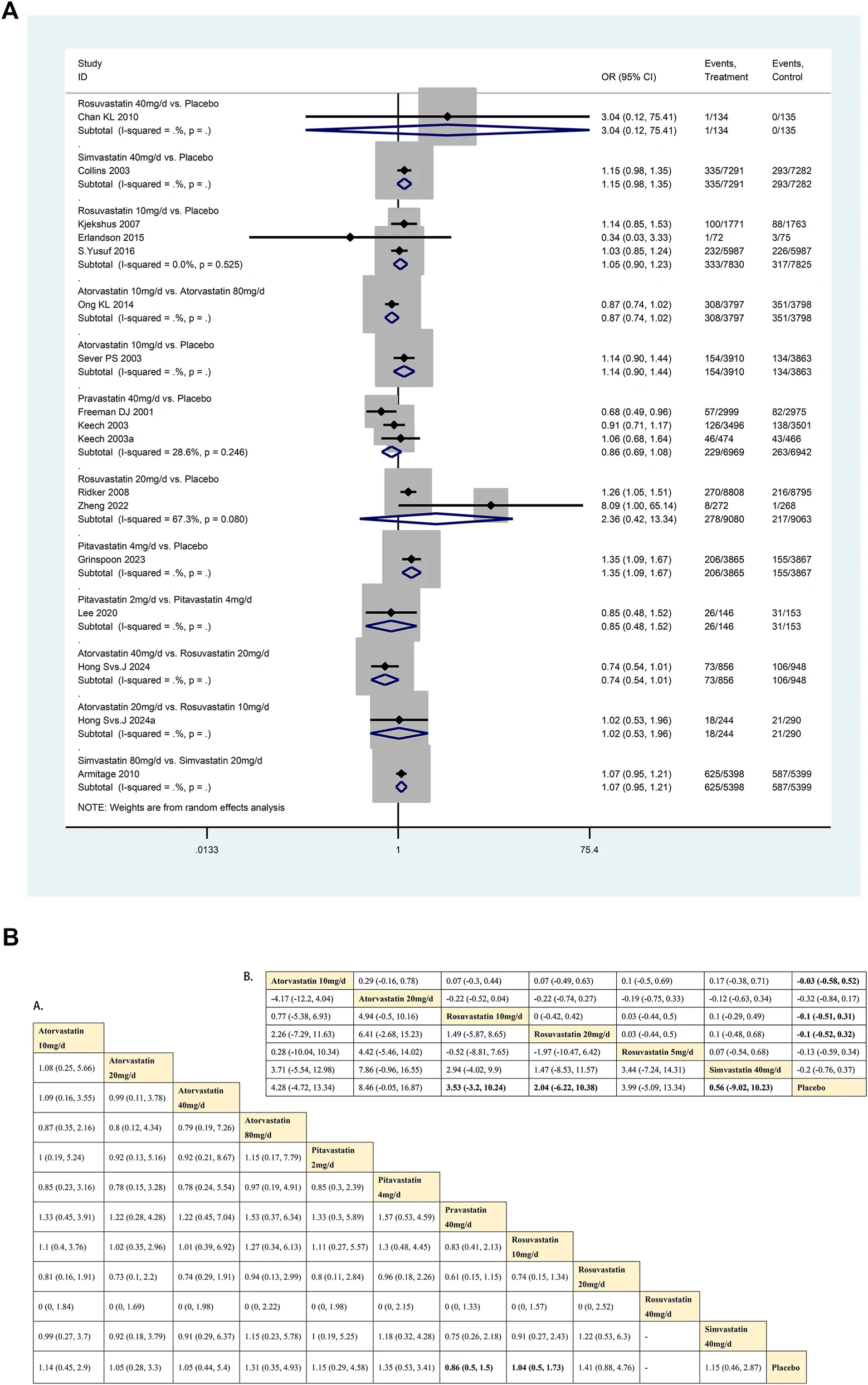
**(A)** Traditional meta-analysis of the risk of the new-onset diabetes mellitus. **(B)** Results of Bayesian network meta-analysis of the outcomes, including the following: **(A)** Risk of new-onset diabetes mellitus analyzed using OR and 95% CrI. **(B)** Upper triangle shows mean difference in HbA1c (%) with MD and 95% CrI, and lower triangle shows mean difference in FPG with MD and 95% CrI. In both triangles, results are reported for the comparison of the column-defining statin versus the row-defining statin. OR, odds ratio; CrI, credible intervals; MD, mean differences; “-” indicates the effect size with very wide credible interval owing to the small sample sizes, which are not shown. All comparisons in the network meta-analysis showed 95% credible intervals that included the null value (1 for OR; 0 for MD), indicating no statistically significant differences.

#### 3.1.4 Secondary outcomes: HbA1c

Eight trials involving 1,465 patients contributed to the analysis of HbA1c in statin-treated versus control groups (Lee et al., 2020; Park et al., 2010; Moutzouri et al., 2011; Koh et al., 2016; Her et al., 2010; Anagnostis et al., 2014; Agrawal et al., 2018; Aberg et al., 2017). Head-to-head comparisons revealed no statistically significant associations between statin doses and HbA1c levels compared to placebo or other statin doses, with one exception—rosuvastatin 10 mg/day significantly reduced HbA1c levels compared with atorvastatin 20 mg/day (MD: −0.20; 95% CI: −0.26 to −0.15) (Supplementary Figure S2A). For the primary NMA, six studies (Park et al., 2010; Moutzouri et al., 2011; Koh et al., 2016; Her et al., 2010; Anagnostis et al., 2014; Agrawal et al., 2018) with a follow-up time of 6–12 weeks formed a connected network comprising rosuvastatin 5, 10, and 20 mg/day, atorvastatin 10 and 20 mg/day, simvastatin 40 mg/day, and placebo. The results demonstrated no significant differences in HbA1c levels between statin doses or versus placebo, however, atorvastatin 10 mg/day showed the smallest numerical increase in HbA1c relative to placebo (MD: 0.03; 95% CrI: −0.52 to 0.58) (Figure 3). According to SUCRA rankings, atorvastatin 10 mg/day was optimal, followed by rosuvastatin 10 mg/day (Supplementary Figure S3B). Additionally, two other studies (Lee et al., 2020; Aberg et al., 2017) formed a network including pitavastatin 2 and 4 mg/day and pravastatin 40 mg/day, and the indirect evidence indicated that although the 95% CrI crossed null, the point estimate of pitavastatin 2 mg/day was lower than for pravastatin 40 mg/day (MD: −0.15; 95% CrI: −0.36 to 0.07). There were insufficient data to assess the consistency analyses and publication bias. Notably, for patients without diabetes, none of the HbA1c exceeded 6.5% during statin management.

#### 3.1.5 Secondary outcomes: FPG levels

Eight trials involving 1,372 patients reported FPG levels of post-treatment (Erlandson et al., 2015; Lee et al., 2020; Park et al., 2010; Moutzouri et al., 2011; Koh et al., 2016; Her et al., 2010; Anagnostis et al., 2014; Aberg et al., 2017). Head-to-head comparisons revealed that rosuvastatin 10 mg/day was associated with lower FPG levels than atorvastatin 20 mg/day (MD: −5.00, 95% CI −6.23 to −3.77), and a similar pattern was observed between pravastatin 40 mg/day and pitavastatin 4 mg/day (MD: 1.80; 95% CI: 1.62–1.98) (Supplementary Figure S2B). Alternatively, an NMA of six RCTs (Erlandson et al., 2015; Park et al., 2010; Moutzouri et al., 2011; Koh et al., 2016; Her et al., 2010; Anagnostis et al., 2014) comparing atorvastatin 10 and 20 mg/day, rosuvastatin 5, 10, and 20 mg/day, simvastatin 40 mg/day, and placebo demonstrated no significant differences between any statin type or dose and placebo (Figure 3). According to SUCRA probabilities, simvastatin 40 mg/day was ranked most favourable with respect to FPG levels, followed by rosuvastatin 20 mg/day and rosuvastatin 10 mg/day (Supplementary Figure S3C). Similarly, indirect evidence from two RCTs (Lee et al., 2020; Aberg et al., 2017) revealed no statistically significant difference between pitavastatin 2 mg/day and pravastatin 40 mg/day (MD: −0.62; 95% CrI: −4.78 to 3.48). Too few studies were included to assess publication bias. The sensitivity analysis excluding high-risk studies yielded results similar to the main analysis.

#### 3.1.6 Secondary outcomes: 2-h postprandial plasma glucose

Only one trial reported changes in 2-h postprandial plasma glucose when comparing rosuvastatin 10 mg/day with placebo (Erlandson et al., 2015). This study found no significant difference between the treatment and placebo groups (P = 0.92).

### 3.2 Disproportionality analysis using the FAERS

From 1 January 2004 to 31 December 2024, 113,562 adverse events (AEs) were reported with statins as the primary suspected drug, of which 7,366 were related to hyperglycemic events (Table 2). Among statin-related adverse event reports, significant disproportionality signals for hyperglycemic events were detected for atorvastatin (n = 5,236/48,391, 10.82%; ROR: 6.11, 95% CI: 5.94–6.29), rosuvastatin (n = 1,341/28,629, 4.68%; ROR: 2.44, 95% CI: 2.31–2.58), and simvastatin (n = 694/29,196, 2.38%; ROR: 1.22, 95% CI: 1.13–1.32). No statistically significant signals were observed for lovastatin (ROR: 1.12, 95% CI: 0.74–1.71), fluvastatin (ROR: 1.22, 95% CI: 0.75–1.96), pitavastatin (ROR: 0.97, 95% CI: 0.48–1.94), or pravastatin (ROR: 0.75, 95% CI: 0.57–1.00). Although not statistically significant, pravastatin showed the lowest reporting frequency (n = 48/4,660, 1.03%) and a comparatively lower ROR estimate than the other statins evaluated. Analyses by event type indicated that hyperglycemic AEs related to atorvastatin were primarily diabetes mellitus (n = 4,654/48,391, 9.62%; ROR: 13.39; 95% CI: 12.98–13.80), whereas mild hyperglycemia was less frequently reported (n = 582/48,391, 1.20%; ROR: 1.04, 95% CI: 0.96–1.13). For all terms of diabetes and additional hyperglycemic conditions, no significant signals were observed for lovastatin, pitavastatin, or pravastatin (Supplementary Table S5).

**TABLE 2:** Disproportionality analysis of hyperglycemic adverse events associated with statins from the FAERS database.

Adverse event category and statistic	Atorvastatin (n = 48,391)	Rosuvastatin (n = 28,629)	Pravastatin (n = 4,660)	Pitavastatin (n = 620)	Simvastatin (n = 29,196)	Fluvastatin (n = 906)	Lovastatin (n = 1,160)
Mild hyperglycemia							
No. of cases, %	582 (1.20%)	375 (1.31%)	39 (0.84%)	4 (0.64%)	483 (1.65%)	7 (0.77%)	18 (1.55%)
ROR (95% CI)	1.04 (0.96–1.13)	1.14 (1.03–1.27)	0.73 (0.53–1.00)	0.67 (0.25–1.78)	1.46 (1.33–1.59)	0.80 (0.38–1.68)	1.37 (0.86–2.19)
Diabetes mellitus							
No. of cases, %	4,654 (9.62%)	966 (3.37%)	9 (0.19%)	4 (0.64%)	211 (0.72%)	10 (1.10%)	4 (0.34%)
ROR (95% CI)	13.39 (12.98–13.80)	4.19 (3.93–4.47)	0.87 (0.45–1.67)	1.75 (0.65–4.67)	0.89 (0.78–1.02)	1.90 (1.02–3.54)	0.62 (0.23–1.65)
Composite hyperglycemic adverse events							
No. of cases, %	5,236 (10.82%)	1,341 (4.68%)	48 (1.03%)	8 (1.29%)	694 (2.38%)	17 (1.88%)	22 (1.89%)
ROR (95% CI)	6.11 (5.94–6.29)	2.44 (2.31–2.58)	0.75 (0.57–1.00)	0.97 (0.48–1.94)	1.22 (1.13–1.32)	1.22 (0.75–1.96)	1.12 (0.74–1.71)

Composite hyperglycemic adverse events outcome includes all terms of diabetes and additional hyperglycemic conditions (SMQ: hyperglycemia/new-onset diabetes mellitus); ROR, reporting odds ratio; CI, confidence interval.

## 4 Discussion

The potential diabetogenic effects of statins remain controversial, particularly with respect to differences across statin types and doses. In the present study, we integrated an NMA of RCTs with a disproportionality analysis of the FAERS to evaluate the glycemic effects of various statin types and doses in patients without baseline diabetes. The NMA demonstrated no statistically significant differences among statins or compared with placebo in the incidence of NODM or in post-treatment HbA1c and FPG levels. Nonetheless, pravastatin 40 mg/day showed a numerical trend toward a lower risk of NODM. In the pharmacovigilance analysis using FAERS, significant disproportionality signals for hyperglycemic events were identified with atorvastatin, rosuvastatin, and simvastatin, whereas no significant disproportionality signal was observed with lovastatin, fluvastatin, pitavastatin, or pravastatin. Consistent with the NMA findings, pravastatin also showed a numerically lower reporting frequency than other statins, suggesting a potentially lower reporting risk for hyperglycemic events. However, in light of the absence of statistical significance and the inherent limitations of both data sources, these findings should be interpreted as hypothesis-generating rather than definitive.

The numerical trends observed for pravastatin may be explained by its hydrophilicity and lower potency. As a highly hepatoselective statin with minimal extrahepatic penetration, pravastatin is less likely to affect β-cell function and insulin secretion (Galicia-Garcia et al., 2020; Lim et al., 2014). Conversely, lipophilic statins such as atorvastatin may more readily penetrate extrahepatic tissues, potentially impairing insulin secretion (Galicia-Garcia et al., 2020; Lim et al., 2014). This interpretation is supported by our FAERS disproportionality sub-analysis, in which the hyperglycemic signal associated with atorvastatin was mainly driven by diabetes mellitus diagnoses rather than mild hyperglycemia, consistent with the proposed mechanistic hypothesis. However, hydrophilicity alone cannot explain why rosuvastatin, although hydrophilic, was associated with a significant hyperglycemic disproportionality signal in the FAERS database and did not demonstrate a lower risk estimate in the NMA. This discrepancy may be partly explained by its relatively high pharmacological potency. High-intensity inhibition of 3-hydroxy-3-methylglutaryl coenzyme A (HMG-CoA) reductase may disrupt intracellular cholesterol homeostasis and impair pancreatic β-cell function, thereby contributing to dysglycemia (Perego et al., 2019; Carmena and Betteridge, 2019; Brandts and Müller-Wieland, 2025). In addition to pharmacological mechanisms, the widespread clinical use of rosuvastatin may have contributed to increased adverse event reporting through the Weber effect. Collectively, statin-associated diabetogenic risk likely reflects the combined effects of lipophilicity, pharmacological potency, and reporting-related factors rather than a single determinant.

The association between statin therapy and NODM has been reported for nearly more than a decade. Previous studies have shown that high-intensity statin therapy is associated with an increased risk of NODM compared to moderate-intensity regimens (Preiss et al., 2011; Collaboration, 2024). In contrast to these prior analyses, the present study focused on individual statin types and specific dose levels, complemented by real-world pharmacovigilance data from the FAERS database. Our NMA did not demonstrate that individual high-intensity statins, such as atorvastatin 40 or 80 mg and rosuvastatin 20 mg, were associated with a significantly higher risk of NODM than other statin regimens. One potential explanation for this discrepancy is that previous studies primarily compared statins according to intensity categories, pooling multiple statins within each category and thereby increasing the effective sample size available for comparison. Such an approach may provide greater statistical power to detect modest differences in NODM risk between intensity groups. In contrast, our NMA evaluated individual statins and dose levels as separate interventions, resulting in smaller sample sizes for specific comparisons. Additionally, the relatively limited number of trials involving statins other than atorvastatin and rosuvastatin, particularly pitavastatin and pravastatin, resulted in a sparse network structure and wide credibility intervals for several indirect comparisons. This may have reduced the precision of effect estimates and increased uncertainty in treatment rankings (Jackson and Turner, 2017; Thorlund and Mills, 2012). FAERS disproportionality analysis identified hyperglycemia-related signals for atorvastatin and rosuvastatin. However, FAERS reflects routine clinical practice, and these signals may be influenced by confounding by indication and channeling bias, as these agents are more frequently prescribed to patients with higher baseline cardiovascular and metabolic risk, which independently increases diabetes risk. In addition, differences in prescribing patterns, drug exposure, and monitoring intensity may contribute to reporting heterogeneity across statins. These findings underscore the need for continued investigation into the individualized diabetogenic risk of statins. As additional evidence becomes available, particularly from head-to-head trials involving statins other than atorvastatin and rosuvastatin, future analyses will be needed to further clarify the comparative glycemic safety of individual statin types and doses.

From a clinical perspective, the present findings suggest that potential differences in glycemic effects across individual statins and doses cannot be excluded. Although no statistically significant differences were observed, the numerical trends in the NMA and the heterogeneous pharmacovigilance signals across statins suggest that potential differences in diabetogenic risk cannot be excluded. Nevertheless, given the well-established cardiovascular benefits of statin therapy, these findings should not be interpreted as a basis for altering guideline-directed statin prescribing (Sattar et al., 2010). Rather, they support the continued importance of glucose monitoring and individualized risk assessment, particularly among patients with prediabetes or other risk factors for diabetes.

## 5 Limitations

This study has several limitations that warrant consideration. First, the definitions of NODM varied across the included RCTs, and most trials were not primarily designed to assess glycemic outcomes, which may have contributed to clinical heterogeneity and reduced the precision of the pooled estimates. Second, the network structure for several outcomes was limited by disconnected comparisons and the absence of closed loops, precluding formal assessments of consistency between direct and indirect evidence. Third, insufficient data were available to perform subgroup analyses based on key patient characteristics, such as HIV status, primary versus secondary prevention, or the presence of heart failure, thereby limiting the generalizability of the findings to these populations. In a fourth limitation, HbA1c reflects average glycemic control over approximately 2–3 months; however, one included study reported a follow-up of only 6 weeks (Park et al., 2010), which may have underestimated the true effects of statins on HbA1c. Finally, the disproportionality analysis based on the FAERS database is subject to the inherent limitations of spontaneous reporting systems, including reporting bias, underreporting, lack of denominator data, and inability to establish causality, which resulted in substantial missing data for important clinical variables (such as dosage information, baseline glycemic status, body mass index, cardiovascular risk, and concomitant medications), thereby precluding adjustment for potential confounding by indication and other patient-level risk factors. Therefore, the ROR-based findings should be interpreted as statistical associations rather than causal relationships.

## 6 Conclusion

This study found no statistically significant differences in glycemic outcomes among individual statins or dose regimens. Although numerical trends suggested a potentially lower diabetogenic risk with pravastatin, the overall evidence remains insufficient to draw definitive conclusions. These findings should be considered hypothesis-generating and warrant further prospective validation.

## Data Availability

The original contributions presented in the study are included in the article/Supplementary Material; further inquiries can be directed to the corresponding authors.
