# Supplementary material for: Risk of hyperglycemia with different statins by type and dose: a network meta-analysis and real-world pharmacovigilance study
Source: Front Pharmacol. 2026 Oct 2;17:1867316. doi: 10.3389/fphar.2026.1867316 (PMC13671962; doi:10.3389/fphar.2026.1867316)
Supplement: Supplementary file 1 [file Supplementaryfile1.docx]

***Supplementary Material***

|  | Low risk | Some concerns | High risk |
| --- | --- | --- | --- |
| Randomization process | 68.2 | 31.8 | 0 |
| Deviations from intended interventions | 95.5 | 4.5 | 0 |
| Mising outcome data | 95.5 | 0 | 4.5 |
| Measurement of the outcome | 100 | 0 | 0 |
| Selection of the reported result | 100 | 0 | 0 |
| Overall Bias | 63.6 | 31.8 | 4.5 |

Figure 1 Summary risk of bias of the included studies. (A) Risk of bias graph. (B) Risk of bias summary.

Figure 2A Traditional meta-analysis of the mean difference in HbA1c (%).

Figure 2B Traditional meta-analysis of the mean difference in fasting plasma glucose (mg/dL).

A.

B.

C.

Figure 3 SUCRA ranking plot of statins. (A) New-onset diabetes mellitus; (B) HbA1c; (C) Fasting plasma glucose. The ranking plot represents the cumulative probability of being the best intervention.

Figure 4 Comparison-adjusted funnel plot for the risk of new-onset diabetes mellitus. (A: Atorvastatin 10mg/d; B: Atorvastatin 20mg/d; C: Atorvastatin 40mg/d; D: Atorvastatin 80mg/d; E: Pitavastatin 2mg/d; F: Pitavastatin 4mg/d; G: Placebo; H: Pravastatin 40mg/d; I: Rosuvastatin 10mg/d; J: Rosuvastatin 20mg/d; K: Rosuvastatin 40mg/d; L: Simvastatin 40mg/d)

**Table 1 PRISMA NMA Checklist of Items to Include When Reporting a Systematic Review Involving a Network Meta-analysis**

| **Section/Topic** | **Item #** | **Checklist Item** | **Reported in Section** |
| --- | --- | --- | --- |
| **TITLE** |  |  |  |
| Title | 1 | Identify the report as a systematic review *incorporating a network meta-analysis (or related form of meta-analysis).* | Title |
|  |  |  |  |
| **ABSTRACT** |  |  |  |
| Structured summary | 2 | Provide a structured summary including, as applicable:  **Background:** main objectives  **Methods:** data sources; study eligibility criteria, participants, and interventions; study appraisal; and *synthesis methods, such as network meta-analysis.*  **Results:** number of studies and participants identified; summary estimates with corresponding confidence/credible intervals; *treatment rankings may also be discussed. Authors may choose to summarize pairwise comparisons against a chosen treatment included in their analyses for brevity.*  **Discussion/Conclusions:** limitations; conclusions and implications of findings.  **Other:** primary source of funding; systematic review registration number with registry name. | Abstract |
| **INTRODUCTION** |  |  |  |
| Rationale | 3 | Describe the rationale for the review in the context of what is already known*, including mention of why a network meta-analysis has been conducted.* | In the Introduction, first paragraph |
| Objectives | 4 | Provide an explicit statement of questions being addressed, with reference to participants, interventions, comparisons, outcomes, and study design (PICOS). | In the Introduction, second paragraph |
| **METHODS** |  |  |  |
| Protocol and registration | 5 | Indicate whether a review protocol exists and if and where it can be accessed (e.g., Web address); and, if available, provide registration information, including registration number. | In Section 2.2 of the Methods |
| Eligibility criteria | 6 | Specify study characteristics (e.g., PICOS, length of follow-up) and report characteristics (e.g., years considered, language, publication status) used as criteria for eligibility, giving rationale. *Clearly describe eligible treatments included in the treatment network, and note whether any have been clustered or merged into the same node (with justification).* | In Section 2.2 of the Methods |
| Information sources | 7 | Describe all information sources (e.g., databases with dates of coverage, contact with study authors to identify additional studies) in the search and date last searched. | In Section 2.2 of the Methods |
| Search | 8 | Present full electronic search strategy for at least one database, including any limits used, such that it could be repeated. | Supplementary  Table 2 |
| Study selection | 9 | State the process for selecting studies (i.e., screening, eligibility, included in systematic review, and, if applicable, included in the meta-analysis). | In Section 2.2 of the Methods  Figure 1 |
| Data collection process | 10 | Describe method of data extraction from reports (e.g., piloted forms, independently, in duplicate) and any processes for obtaining and confirming data from investigators. | In Section 2.2 of the Methods |
| Data items | 11 | List and define all variables for which data were sought (e.g., PICOS, funding sources) and any assumptions and simplifications made. | In Section 2.3 of the Methods  Table 1 |
| **Geometry of the network** | **S1** | Describe methods used to explore the geometry of the treatment network under study and potential biases related to it. This should include how the evidence base has been graphically summarized for presentation, and what characteristics were compiled and used to describe the evidence base to readers. | In Section 2.3 of the Methods |
| Risk of bias within individual studies | 12 | Describe methods used for assessing risk of bias of individual studies (including specification of whether this was done at the study or outcome level), and how this information is to be used in any data synthesis. | In Section 2.3 of the Methods |
| Summary measures | 13 | State the principal summary measures (e.g., risk ratio, difference in means). *Also describe the use of additional summary measures assessed, such as treatment rankings and surface under the cumulative ranking curve (SUCRA) values, as well as modified approaches used to present summary findings from meta-analyses.* | In Section 2.3 of the Methods |
| Planned methods of analysis | 14 | Describe the methods of handling data and combining results of studies for each network meta-analysis. This should include, but not be limited to:   - *Handling of multi-arm trials;* - *Selection of variance structure;* - *Selection of prior distributions in Bayesian analyses; and* - *Assessment of model fit.* | In Section 2.3 of the Methods |
| **Assessment of Inconsistency** | **S2** | Describe the statistical methods used to evaluate the agreement of direct and indirect evidence in the treatment network(s) studied. Describe efforts taken to address its presence when found. | In Section 2.3 of the Methods |
| Risk of bias across studies | 15 | Specify any assessment of risk of bias that may affect the cumulative evidence (e.g., publication bias, selective reporting within studies). | In Section 2.3 of the Methods |
| Additional analyses | 16 | Describe methods of additional analyses if done, indicating which were pre-specified. This may include, but not be limited to, the following:   - Sensitivity or subgroup analyses; - Meta-regression analyses; - *Alternative formulations of the treatment network; and* - *Use of alternative prior distributions for Bayesian analyses (if applicable).* | In Section 2.3 of the Methods |
| **RESULTS†** |  |  |  |
| Study selection | 17 | Give numbers of studies screened, assessed for eligibility, and included in the review, with reasons for exclusions at each stage, ideally with a flow diagram. | In Section 3.1.1 of the Results  Figure 1 |
| **Presentation of network structure** | **S3** | Provide a network graph of the included studies to enable visualization of the geometry of the treatment network. | Figure 2 |
| **Summary of network geometry** | **S4** | Provide a brief overview of characteristics of the treatment network. This may include commentary on the abundance of trials and randomized patients for the different interventions and pairwise comparisons in the network, gaps of evidence in the treatment network, and potential biases reflected by the network structure. | In Section 3.1.1 of the Results  Figure 2 |
| Study characteristics | 18 | For each study, present characteristics for which data were extracted (e.g., study size, PICOS, follow-up period) and provide the citations. | Table 1 |
| Risk of bias within studies | 19 | Present data on risk of bias of each study and, if available, any outcome level assessment. | In Section 3.1.2 of the Results  Supplementary  Material Figure 1 |
| Results of individual studies | 20 | For all outcomes considered (benefits or harms), present, for each study: 1) simple summary data for each intervention group, and 2) effect estimates and confidence intervals. *Modified approaches may be needed to deal with information from larger networks.* | In Sections 3.1.3–3.1.5 of the Results |
| Synthesis of results | 21 | Present results of each meta-analysis done, including confidence/credible intervals. *In larger networks, authors may focus on comparisons versus a particular comparator (e.g. placebo or standard care), with full findings presented in an appendix. League tables and forest plots may be considered to summarize pairwise comparisons.* If additional summary measures were explored (such as treatment rankings), these should also be presented. | In Sections 3.1.3–3.1.5 of the Results Figure 3  Supplementary  Material Figure 2A, Figure 2B and Figure 3 |
| **Exploration for inconsistency** | **S5** | Describe results from investigations of inconsistency. This may include such information as measures of model fit to compare consistency and inconsistency models, *P* values from statistical tests, or summary of inconsistency estimates from different parts of the treatment network. | In Sections 3.1.3–3.1.5 of the Results |
| Risk of bias across studies | 22 | Present results of any assessment of risk of bias across studies for the evidence base being studied. | In Section 3.1.2 of the Results  Supplementary  Material Figure 1 |
| Results of additional analyses | 23 | Give results of additional analyses, if done (e.g., sensitivity or subgroup analyses, meta-regression analyses*, alternative network geometries studied, alternative choice of prior distributions for Bayesian analyses,* and so forth). | In Section 3.1.3 of the Results |
| **DISCUSSION** |  |  |  |
| Summary of evidence | 24 | Summarize the main findings, including the strength of evidence for each main outcome; consider their relevance to key groups (e.g., healthcare providers, users, and policy-makers). | In the Discussion |
| Limitations | 25 | Discuss limitations at study and outcome level (e.g., risk of bias), and at review level (e.g., incomplete retrieval of identified research, reporting bias). *Comment on the validity of the assumptions, such as transitivity and consistency. Comment on any concerns regarding network geometry (e.g., avoidance of certain comparisons).* | In the Limitations |
| Conclusions | 26 | Provide a general interpretation of the results in the context of other evidence, and implications for future research. | In the Conclusions |
| **FUNDING** |  |  |  |
| Funding | 27 | Describe sources of funding for the systematic review and other support (e.g., supply of data); role of funders for the systematic review. This should also include information regarding whether funding has been received from manufacturers of treatments in the network and/or whether some of the authors are content experts with professional conflicts of interest that could affect use of treatments in the network. | In the Funding |

**Table 2 Literature search strategy**

| **PubMed** | |
| --- | --- |
| 1 | ((Hydroxymethylglutaryl-CoA Reductase Inhibitors[MeSH Terms]) OR (Statin) OR (Statins[Text Word]) OR (Hydroxymethylglutaryl CoA Reductase Inhibitors[Text Word]) OR (Inhibitors, Hydroxymethylglutaryl-CoA Reductase[Text Word]) OR (Reductase Inhibitors, Hydroxymethylglutaryl-CoA[Text Word]) OR (HMG CoA Reductase Inhibitor[Text Word]) OR (Inhibitors, HMG-CoA Reductase[Text Word]) OR (Reductase Inhibitors, HMG-CoA[Text Word]) OR (Inhibitors, Hydroxymethylglutaryl Coenzyme A [Text Word]) OR (Hydroxymethylglutaryl-CoA Reductase Inhibitor[Text Word]) OR (HMG-CoA Statins[Text Word]) OR (Hydroxymethylglutaryl-CoA Inhibitors[Text Word])) OR (atorvastatin OR fluvastatin OR lovastatin OR pitavastatin OR pravastatin OR rosuvastatin OR simvastatin OR lipitor OR lescol OR mevacor OR livalo OR pravachol OR crestor OR zocor) |
| 2 | (((Diabetes Mellitus[MeSH Terms]) OR (Hyperglycemia[MeSH Terms]) OR (Diabetes Mellitus) OR (Diabetes) OR Diabetic OR (DM) OR (Diabetes Mellitus, Type 1) OR (Diabetes Mellitus, Type 2) OR Hyperglycaemia OR (Hyperglycemia)) AND ((new onset) OR (new-onset) OR (incident) OR (risk) OR (newly diagnosed) OR (transient) OR (incidence) OR (rate))) OR (Glycosylated haemoglobin abnormal) OR (Glycosylated haemoglobin increased) OR (Insulin resistance) |
| 3 | ((((((randomized controlled trial [Publication Type]) OR (controlled clinical trial [Publication Type])) OR (random*[Title/Abstract])) OR (placebo [Title/Abstract])) OR (trial [Title/Abstract])) OR (clinical trials as topic [MeSH Terms])) NOT ((animals [MeSH Terms]) NOT (humans [MeSH Terms])) |
| 4 | #1 AND #2 AND #3 |
| **Embase** | |
| 1 | 'hydroxymethylglutaryl coenzyme a reductase inhibitor'/exp OR 'hydroxymethylglutaryl coenzyme a reductase inhibitor' OR statin OR 'hydroxymethylglutaryl coa reductase inhibitors' OR 'hmg coa reductase inhibitor' OR 'hydroxymethylglutaryl-coa reductase inhibitor' OR 'hmg-coa statins' OR 'hydroxymethylglutaryl-coa inhibitors' OR atorvastatin OR fluvastatin OR lovastatin OR pitavastatin OR pravastatin OR rosuvastatin OR simvastatin OR lipitor OR lescol OR mevacor OR livalo OR pravachol OR crestor OR zocor |
| 2 | 'new onset' OR 'new-onset' OR 'incident' OR 'incidence' OR risk OR 'newly diagnosed' OR transient OR rate |
| 3 | 'diabetes mellitus'/exp OR 'hyperglycemia'/exp OR 'diabetes mellitus' OR 'hyperglycemia' OR diabetes OR diabetic OR 'non insulin dependent diabetes mellitus' OR 'insulin dependent diabetes mellitus' |
| 4 | 'glycosylated haemoglobin abnormal' OR 'glycosylated haemoglobin increased' OR 'insulin resistance' |
| 5 | #2 OR #3 OR #4 |
| 6 | 'clinical trial'/exp AND topic OR 'randomized controlled trial':it OR 'controlled clinical trial':it OR randomized:ab,ti OR placebo:ab,ti OR randomly:ab,ti OR trial:ab,ti |
| 7 | #1 AND #5 AND #6 |
| **Cochrane Library** | |
| 1 | MeSH descriptor: [Hydroxymethylglutaryl-CoA Reductase Inhibitors] explode all trees |
| 2 | "Hydroxymethylglutaryl-CoA Reductase Inhibitors" OR statin OR "hydroxymethylglutaryl coa reductase inhibitors" OR "hmg coa reductase inhibitor" OR "hydroxymethylglutaryl-coa reductase inhibitor" OR "hmg-coa statins" OR "hydroxymethylglutaryl-coa inhibitors" OR "atorvastatin OR fluvastatin OR lovastatin OR pitavastatin OR pravastatin OR rosuvastatin OR simvastatin OR lipitor OR lescol OR mevacor OR livalo OR pravachol OR crestor OR zocor |
| 3 | #1 OR #2 |
| 4 | "new onset" OR "new-onset" OR "incident" OR "incidence" OR risk OR "newly diagnosed" OR transient OR rate |
| 5 | MeSH descriptor: [Diabetes Mellitus] explode all trees |
| 6 | MeSH descriptor: [Hyperglycemia] explode all trees |
| 7 | "diabetes mellitus" OR "hyperglycemia" OR "diabetes mellitus" OR "hyperglycemia" OR diabetes OR diabetic OR "non insulin dependent diabetes mellitus" OR "insulin dependent diabetes mellitus" |
| 8 | "glycosylated haemoglobin abnormal" OR "glycosylated haemoglobin increased" OR "insulin resistance" |
| 9 | #5 OR #7 |
| 10 | #9 AND #4 |
| 11 | #10 OR #6 OR #8 |
| 12 | MeSH descriptor: [Clinical Trial] explode all trees |
| 13 | MeSH descriptor: [Randomized Controlled Trial] explode all trees |
| 14 | MeSH descriptor: [Controlled Clinical Trial] explode all trees |
| 15 | randomized OR placebo OR randomly OR trial |
| 16 | #12 OR #13 OR #14 OR #15 |
| 17 | #3 AND #11 AND #16 |

**Table 3 Classification of hyperglycemic adverse events terms.**

| **Hyperglycemic disorder sub-category** | **MedDRA Preferred Terms (PT) included** |
| --- | --- |
| Mild hyperglycemia | Hyperglycaemia |
|  | Impaired fasting glucose |
|  | Insulin resistance |
|  | Glucose tolerance impaired |
|  | Blood glucose increased |
|  | Glycated albumin increased |
|  | Glycosylated haemoglobin abnormal |
|  | Glycosylated haemoglobin increased |
|  | fructosamine increased |
|  | Glucose urine present |
|  | Glycosuria |
| Diabetes mellitus | Acquired lipoatrophic diabetes |
|  | Diabetes mellitus |
|  | Diabetes mellitus inadequate control |
|  | Fulminant type 1 diabetes mellitus |
|  | Diabetes with hyperosmolarity |
|  | Insulin resistant diabetes |
|  | Insulin-requiring type 2 diabetes mellitus |
|  | Ketosis-prone diabetes mellitus |
|  | Latent autoimmune diabetes in adults |
|  | Monogenic diabetes |
|  | Pancreatogenous diabetes |
|  | Type 1 diabetes mellitus |
|  | Type 2 diabetes mellitus |
|  | Diabetic arteritis |
|  | Diabetic coronary microangiopathy |
|  | Diabetic ketoacidotic hyperglycaemic coma |
|  | Diabetic hyperglycaemic coma |
|  | Diabetic hyperosmolar coma |
|  | Diabetic coma |
|  | Hyperglycaemic hyperosmolar nonketotic syndrome |
|  | Diabetic ketoacidosis |
|  | Diabetic ketosis |
|  | Diabetic metabolic decompensation |
|  | Euglycaemic diabetic ketoacidosis |
|  | Diabetic hepatopathy |
|  | Diabetic wound |
|  | Hyperglycaemic seizure |
|  | Hyperglycaemic unconsciousness |
|  | Ketoacidosis |
|  | Ketosis |
|  | Ketonuria |
|  | Urine ketone body present |

MedDRA Preferred Terms of the SMQ- ”hyperglycemic/new-onset diabetes mellitus” were subcategorized into mild hyperglycemia and diabetes. The table presents the preferred terms (PTs) included in each subcategory (mild hyperglycemic disorders and diabetes mellitus).

**Table 4 Results of the meta-regression analysis on the risk of NODM.**

|  | **Ages** | **Female ratio** | **Follow-up time** |
| --- | --- | --- | --- |
| Coef. | 0.017 | 0.005 | -0.032 |
| Std. Err. | 0.011 | 0.004 | 0.039 |
| *p* >\|t\| | 0.16 | 0.26 | 0.43 |

Coef., coefficient; Std. Err., standard error; NODM: new-onset diabetes mellitus.

**Table 5. Disproportionality analysis of different type of hyperglycaemic adverse events associated with statins** **from the FAERS database.**

| **Drug** | **Hyperglycemic disorder sub-category** | **Preferred term (PT)** | **is ADR** | **No. of cases** | **ROR (95%CI)** | **PRR** | **Chi squared** |
| --- | --- | --- | --- | --- | --- | --- | --- |
| **Atorvastatin** | Mild hyperglycaemia | Glycosylated haemoglobin increased | Yes | 114 | 2.16 (1.80-2.60) | 2.159 | 69.255 |
|  |  | Glucose tolerance impaired | Yes | 64 | 6.06 (4.73-7.76) | 6.053 | 259.2 |
|  |  | Blood glucose increased | No | 343 | 0.83 (0.74-0.92) | 0.829 | 11.917 |
|  |  | Hyperglycaemia | No | 50 | 0.70 (0.53-0.93) | 0.703 | 5.948 |
|  |  | Glycosylated haemoglobin abnormal | No | 4 | 1.44 (0.54-3.84) | 1.436 | 0.182 |
|  |  | Glycosuria | No | 3 | 1.07 (0.34-3.32) | 1.069 | 0.034 |
|  |  | Glucose urine present | No | 2 | 0.86 (0.21-3.43) | 0.856 | 0.012 |
|  |  | Insulin resistance | No | 2 | 0.44 (0.11-1.78) | 0.444 | 0.887 |
|  | Diabetes mellitus | Type 2 diabetes mellitus | Yes | 4011 | 77.85 (75.10-80.70) | 71.481 | 221097.1 |
|  |  | Diabetes mellitus | Yes | 528 | 3.00 (2.76-3.27) | 2.982 | 688.83 |
|  |  | Diabetic metabolic decompensation | Yes | 10 | 3.94 (2.11-7.35) | 3.939 | 18.805 |
|  |  | Diabetes mellitus inadequate control | No | 41 | 1.10 (0.81-1.50) | 1.102 | 0.29 |
|  |  | Insulin resistant diabetes | No | 1 | 3.49 (0.49-25.12) | 3.494 | 0.155 |
|  |  | Insulin-requiring type 2 diabetes mellitus | No | 2 | 3.15 (0.78-12.70) | 3.151 | 1.161 |
|  |  | Type 1 diabetes mellitus | No | 15 | 1.12 (0.68-1.87) | 1.125 | 0.101 |
|  |  | Diabetic hyperosmolar coma | No | 1 | 1.04 (0.15-7.41) | 1.04 | 0.222 |
|  |  | Diabetic coma | No | 13 | 1.73 (1.00-2.99) | 1.732 | 3.299 |
|  |  | Hyperglycaemic hyperosmolar nonketotic syndrome | No | 6 | 2.33 (1.04-5.20) | 2.33 | 3.288 |
|  |  | Diabetic ketoacidosis | No | 17 | 0.29 (0.18-0.47) | 0.293 | 28.3 |
|  |  | Ketoacidosis | No | 3 | 0.15 (0.05-0.45) | 0.147 | 14.031 |
|  |  | Ketonuria | No | 2 | 1.07 (0.27-4.29) | 1.069 | 0.074 |
|  |  | Urine ketone body present | No | 4 | 1.29 (0.48-3.44) | 1.287 | 0.049 |
| **Rosuvastatin** | Mild hyperglycaemia | Glucose tolerance impaired | Yes | 29 | 4.59 (3.18-6.62) | 4.587 | 77.042 |
|  |  | Blood glucose increased | No | 243 | 0.99 (0.88-1.13) | 0.994 | 0.004 |
|  |  | Glycosylated haemoglobin increased | No | 61 | 1.95 (1.52-2.51) | 1.948 | 27.116 |
|  |  | Hyperglycaemia | No | 33 | 0.78 (0.56-1.10) | 0.785 | 1.728 |
|  |  | Glucose urine present | No | 4 | 2.91 (1.09-7.77) | 2.906 | 3.253 |
|  |  | Insulin resistance | No | 4 | 1.51 (0.56-4.02) | 1.507 | 0.267 |
|  |  | Impaired fasting glucose | No | 1 | 3.55 (0.50-25.39) | 3.549 | 0.166 |
|  | Diabetes mellitus | Type 2 diabetes mellitus | Yes | 527 | 13.17 (12.07-14.37) | 12.949 | 5648.71 |
|  |  | Diabetes mellitus | Yes | 376 | 3.62 (3.27-4.01) | 3.584 | 695.113 |
|  |  | Diabetes mellitus inadequate control | No | 27 | 1.23 (0.84-1.79) | 1.227 | 0.913 |
|  |  | Type 1 diabetes mellitus | No | 14 | 1.78 (1.05-3.00) | 1.777 | 3.991 |
|  |  | Diabetic ketoacidosis | No | 6 | 0.17 (0.08-0.39) | 0.175 | 22.547 |
|  |  | Diabetic coma | No | 2 | 0.45 (0.11-1.79) | 0.449 | 0.858 |
|  |  | Ketoacidosis | No | 5 | 0.41 (0.17-1.00) | 0.414 | 3.572 |
|  |  | Euglycaemic diabetic ketoacidosis | No | 4 | 0.73 (0.27-1.95) | 0.732 | 0.169 |
|  |  | Pancreatogenous diabetes | No | 2 | 38.45 (9.09-162.71) | 38.449 | 37.151 |
|  |  | Diabetic metabolic decompensation | No | 1 | 0.66 (0.09-4.68) | 0.658 | 0.000 |
|  |  | Hyperglycaemic hyperosmolar nonketotic syndrome | No | 1 | 0.65 (0.09-4.64) | 0.653 | 0.001 |
|  |  | Urine ketone body present | No | 1 | 0.54 (0.08-3.86) | 0.543 | 0.063 |
| **Pravastatin** | Mild hyperglycaemia | Blood glucose increased | No | 23 | 0.58 (0.38-0.87) | 0.578 | 6.732 |
|  |  | Hyperglycaemia | No | 7 | 1.02 (0.49-2.15) | 1.024 | 0.017 |
|  |  | Glycosylated haemoglobin increased | No | 7 | 1.37 (0.65-2.88) | 1.371 | 0.38 |
|  |  | Insulin resistance | No | 1 | 2.31 (0.32-16.44) | 2.312 | 0.01 |
|  |  | Glucose tolerance impaired | No | 1 | 0.96 (0.14-6.85) | 0.964 | 0.207 |
|  | Diabetes mellitus | Type 2 diabetes mellitus | No | 5 | 0.73 (0.31-1.77) | 0.736 | 0.248 |
|  |  | Diabetes mellitus inadequate control | No | 4 | 1.12 (0.42-2.97) | 1.116 | 0.002 |
| **Pitavastatin** | Mild hyperglycaemia | Blood glucose increased | No | 3 | 0.56 (0.18-1.75) | 0.567 | 0.614 |
|  |  | Glycosylated haemoglobin increased | No | 1 | 1.47 (0.21-10.47) | 1.472 | 0.047 |
|  | Diabetes mellitus | Diabetes mellitus | No | 3 | 1.31 (0.42-4.09) | 1.313 | 0.02 |
|  |  | Diabetic hyperglycaemic coma | No | 1 | 111.96 (15.66-800.19) | 111.782 | 26.812 |
| **Simvastatin** | Mild hyperglycaemia | Glucose tolerance impaired | Yes | 22 | 3.41 (2.24-5.18) | 3.404 | 34.725 |
|  |  | Insulin resistance | Yes | 7 | 2.59 (1.23-5.45) | 2.592 | 5.31 |
|  |  | Blood glucose increased | No | 390 | 1.57 (1.42-1.74) | 1.566 | 79.66 |
|  |  | Hyperglycaemia | No | 36 | 0.84 (0.61-1.17) | 0.84 | 0.94 |
|  |  | Glycosylated haemoglobin increased | No | 27 | 0.84 (0.58-1.23) | 0.843 | 0.635 |
|  |  | Impaired fasting glucose | No | 1 | 3.48 (0.49-24.89) | 3.48 | 0.155 |
|  | Diabetes mellitus | Diabetes mellitus | No | 114 | 1.06 (0.88-1.27) | 1.06 | 0.327 |
|  |  | Type 2 diabetes mellitus | No | 55 | 1.29 (0.99-1.69) | 1.293 | 3.351 |
|  |  | Diabetes mellitus inadequate control | No | 22 | 0.98 (0.65-1.49) | 0.98 | 0.00 |
|  |  | Diabetic ketoacidosis | No | 9 | 0.26 (0.13-0.50) | 0.257 | 18.563 |
|  |  | Type 1 diabetes mellitus | No | 4 | 0.50 (0.19-1.32) | 0.496 | 1.569 |
|  |  | Ketoacidosis | No | 2 | 0.16 (0.04-0.65) | 0.162 | 7.82 |
|  |  | Diabetic hyperosmolar coma | No | 1 | 1.73 (0.24-12.31) | 1.727 | 0.011 |
|  |  | Diabetic coma | No | 1 | 0.22 (0.03-1.56) | 0.22 | 2.041 |
|  |  | Diabetic metabolic decompensation | No | 1 | 0.65 (0.09-4.59) | 0.645 | 0.002 |
|  |  | Insulin-requiring type 2 diabetes mellitus | No | 1 | 2.60 (0.36-18.56) | 2.6 | 0.034 |
|  |  | Urine ketone body present | No | 1 | 0.53 (0.08-3.78) | 0.532 | 0.076 |
| **Fluvastatin** | Mild hyperglycaemia | Glycosylated haemoglobin increased | Yes | 4 | 4.04 (1.51-10.80) | 4.029 | 6.337 |
|  |  | Blood glucose increased | No | 3 | 0.39 (0.12-1.20） | 0.388 | 2.34 |
|  | Diabetes mellitus | Diabetes mellitus | No | 7 | 2.11 (1.00-4.43) | 2.097 | 3.005 |
|  |  | Type 1 diabetes mellitus | No | 1 | 4.01 (0.56-28.49) | 4.004 | 0.251 |
|  |  | Type 2 diabetes mellitus | No | 1 | 0.78 (0.11-5.38) | 0.757 | 0.024 |
|  |  | Ketoacidosis | No | 1 | 2.62 (0.37-18.65) | 2.622 | 0.037 |
| **Lovastatin** | Mild hyperglycaemia | Blood glucose increased | No | 14 | 1.42 (0.84-2.40) | 1.413 | 1.315 |
|  |  | Hyperglycaemia | No | 2 | 1.17 (0.29-4.71) | 1.175 | 0.024 |
|  |  | Glycosylated haemoglobin increased | No | 1 | 0.79 (0.11-5.59) | 0.787 | 0.041 |
|  |  | Glucose tolerance impaired | No | 1 | 3.88 (0.54-27.56) | 3.875 | 0.227 |
|  | Diabetes mellitus | Diabetes mellitus | No | 2 | 0.47 (0.12-1.87) | 0.468 | 0.739 |
|  |  | Ketoacidosis | No | 1 | 2.05 (0.29-14.56) | 2.048 | 0.00 |
|  |  | Type 2 diabetes mellitus | No | 1 | 0.59 (0.08-4.20) | 0.591 | 0.022 |
